# LipoAssist: a structured GPT-4–based clinical workflow for preliminary lipedema assessment

**DOI:** 10.3389/fmed.2026.1909592

**Published:** 2026-08-31

**Authors:** Ozkan Yukselmis, Serpil Demirulus, İsmail Gunes Gokmen, Hudanur Coskun

**Affiliations:** 1 Department of Physical Medicine and Rehabilitation, Diyarbakir Gazi Yasargil Training and Research Hospital, Diyarbakir, Türkiye; 2 Department of Physical Medicine and Rehabilitation, Yavuz Selim Bone Diseases and Rehabilitation Hospital, Trabzon, Türkiye; 3 Department of Physical Medicine and Rehabilitation, Eskisehir City Hospital, Eskisehir, Türkiye; 4 Department of Physical Medicine and Rehabilitation, Safranbolu State Hospital, Karabuk, Türkiye

**Keywords:** artificial intelligence, clinical workflow, GPT-4, large language models, lipedema, medical history taking, simulation study

## Abstract

**Objective:**

Lipedema is a chronic adipose tissue disorder characterized by bilateral and symmetrical subcutaneous fat accumulation, predominantly affecting women. Because it is frequently confused with obesity and lymphedema, diagnosis may be delayed. This proof-of-concept study aimed to evaluate the feasibility of LipoAssist, a structured GPT-4–based clinical workflow designed for the preliminary assessment of lipedema under simulated conditions.

**Methods:**

Ten simulated clinical scenarios representing lipedema and relevant differential diagnoses were evaluated using LipoAssist. The workflow was designed to obtain a structured medical history, assess clinically relevant symptoms, and generate a standardized case summary. Three board-certified Physical Medicine and Rehabilitation specialists independently evaluated the AI-generated outputs using a 5-point Likert scale across six criteria. A total of 180 ratings were analyzed. Inter-rater agreement was assessed using the intraclass correlation coefficient and Kendall’s coefficient of concordance.

**Results:**

The overall mean performance score was 3.55 ± 0.64. The highest scores were observed for correct understanding of the clinical condition (4.63 ± 0.49) and identification of the most likely diagnosis (4.53 ± 0.51). Lower scores were recorded for recommendations regarding further diagnostic evaluation (2.17 ± 0.83) and assessment of surgical necessity (1.93 ± 0.64). Case-summary clarity and adequacy of history taking received mean scores of 3.87 ± 0.63 and 3.73 ± 0.64, respectively. Inter-rater agreement was good (ICC = 0.82; 95% CI: 0.68–0.91), and Kendall’s W was 0.79.

**Conclusion:**

LipoAssist demonstrated promising performance in structured history taking, organization of clinical information, and identification of the most likely diagnosis in simulated lipedema scenarios. However, its performance was limited in advanced diagnostic recommendations and surgical decision-making. These findings support the feasibility of a structured GPT-4–based workflow under simulated conditions but do not establish clinical validity, diagnostic accuracy, or readiness for routine implementation.

## 1 Introduction

Lipedema is a chronic adipose tissue disorder characterized by symmetrical fat accumulation in the lower extremities, predominantly affecting women. The condition typically manifests during periods of hormonal fluctuation such as puberty, pregnancy, or menopause, and is associated with pain, tenderness, easy bruising, and functional impairment. Lipedema is frequently confused with obesity and lymphedema, which may result in delayed diagnosis and difficulties in accessing appropriate treatment. Therefore, early diagnosis and accurate clinical guidance are of considerable importance (1–3).

In recent years, large language models (LLMs) have emerged as promising tools for clinical decision support. Systems such as ChatGPT can assist with structured medical history taking, organization of clinical information, and prioritization of possible diagnoses through natural language processing, thereby supporting both clinicians and patients (4–8).

Despite these advances, the clinical reliability of LLMs remains under active investigation. Although previous studies have shown that ChatGPT can provide useful medical information across various specialties, its performance may decline when complex diagnostic reasoning or advanced therapeutic recommendations are required (9–14).

Evidence regarding the application of LLMs in lipedema remains limited. Leypold et al. evaluated a GPT-4–based consultation assistant for lipedema and concluded that, although the model may support patient counseling, caution is warranted when using AI-generated recommendations for clinical decision-making (15). However, that study primarily focused on patient counseling and did not include a structured criterion-based evaluation of AI performance across clinically representative scenarios.

Therefore, the present study aimed to evaluate a structured GPT-4–based clinical workflow, referred to as LipoAssist, for the preliminary assessment of lipedema through simulated clinical scenarios and expert evaluation by specialists in Physical Medicine and Rehabilitation (PM&R). LipoAssist does not represent a newly developed large language model or an independently trained artificial intelligence system. Rather, it consists of a structured prompt engineering framework designed to standardize patient history taking, symptom evaluation, differential diagnosis guidance, and presentation of a structured clinical summary using the capabilities of GPT-4. Accordingly, this study evaluates the performance of a workflow built upon an existing large language model rather than the development of a novel foundation AI model.

## 2 Materials and methods

### 2.1 Design of the artificial intelligence–based clinical assistant

In this study, a structured GPT-4–based clinical workflow, designated LipoAssist, was designed to support the preliminary assessment of patients with suspected lipedema. LipoAssist is not an independently developed artificial intelligence model; instead, it is a prompt-engineered workflow built upon the GPT-4 large language model. The workflow was designed to standardize the process of patient history taking, symptom assessment, differential diagnosis support, and structured case summarization. No additional machine-learning algorithms, proprietary reasoning engines, retrieval-augmented generation (RAG), fine-tuning procedures, external knowledge bases, or expert-system architectures were incorporated into the workflow.

The workflow was implemented using the ChatGPT web interface (OpenAI, San Francisco, CA, USA) and was based on the GPT-4 architecture available to ChatGPT users during the study period. The study did not use the OpenAI API or any custom software environment. No fine-tuning, retrieval-augmented generation (RAG), external knowledge bases, plugins, or additional computational modules were incorporated. All interactions were performed using the same ChatGPT environment and identical prompt instructions.

**Figure 1:**
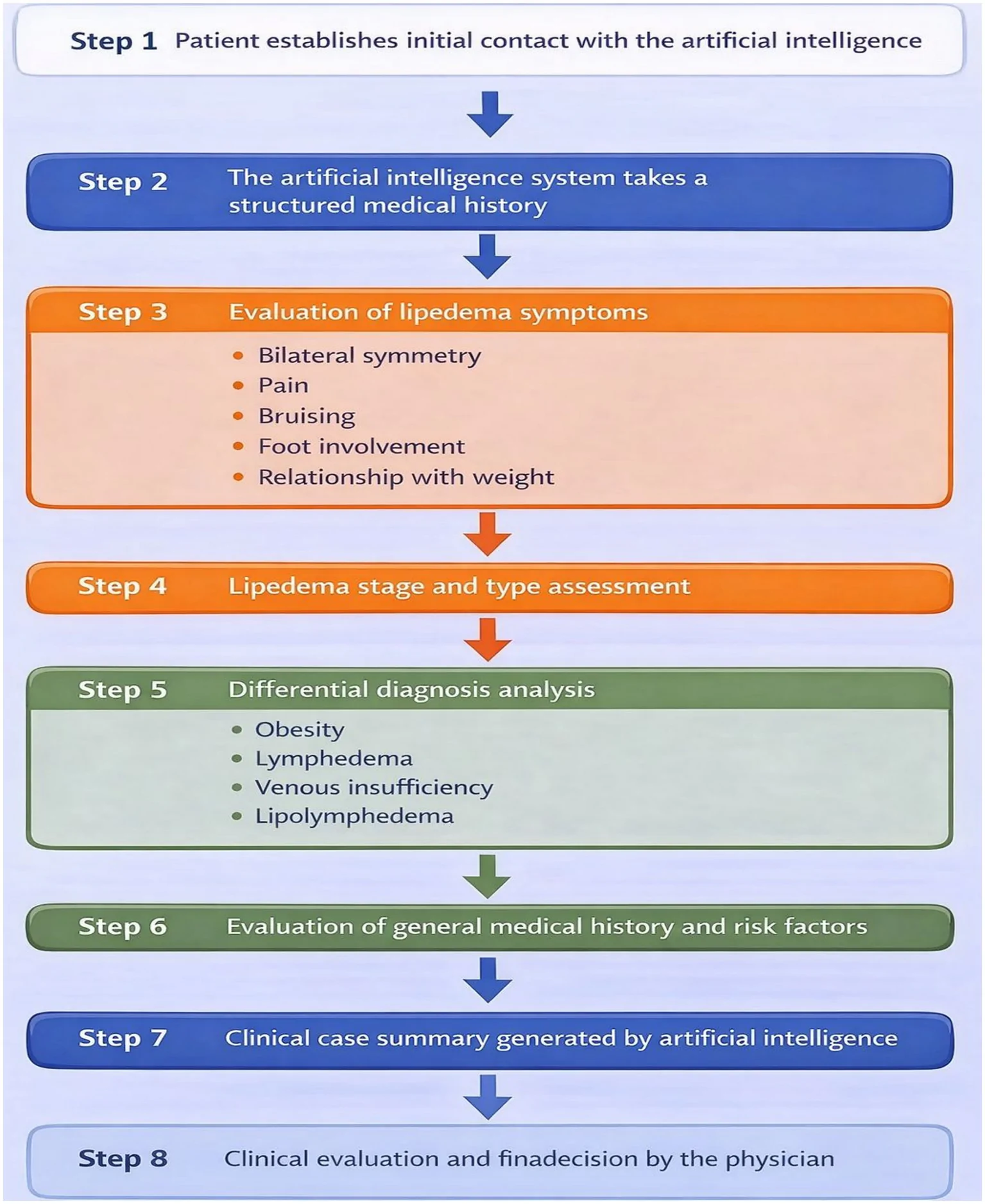
The LipoAssist-based preliminary assessment algorithm for patients with suspected lipedema.

Accordingly, the novelty of LipoAssist lies in the design of its structured clinical workflow and prompt engineering strategy rather than in the development of a new artificial intelligence model. GPT-4 functions as the underlying language model, whereas LipoAssist defines the standardized interaction sequence, clinical questioning strategy, organization of patient information, and structured presentation of findings to the physician (Figure 1).

The primary objective of LipoAssist is not to establish a diagnosis, but to support the clinical decision-making process, systematically collect patient information in a structured manner, and enable the physician to begin the examination more prepared.

### 2.2 Patient interview phase

**Figure 2:**
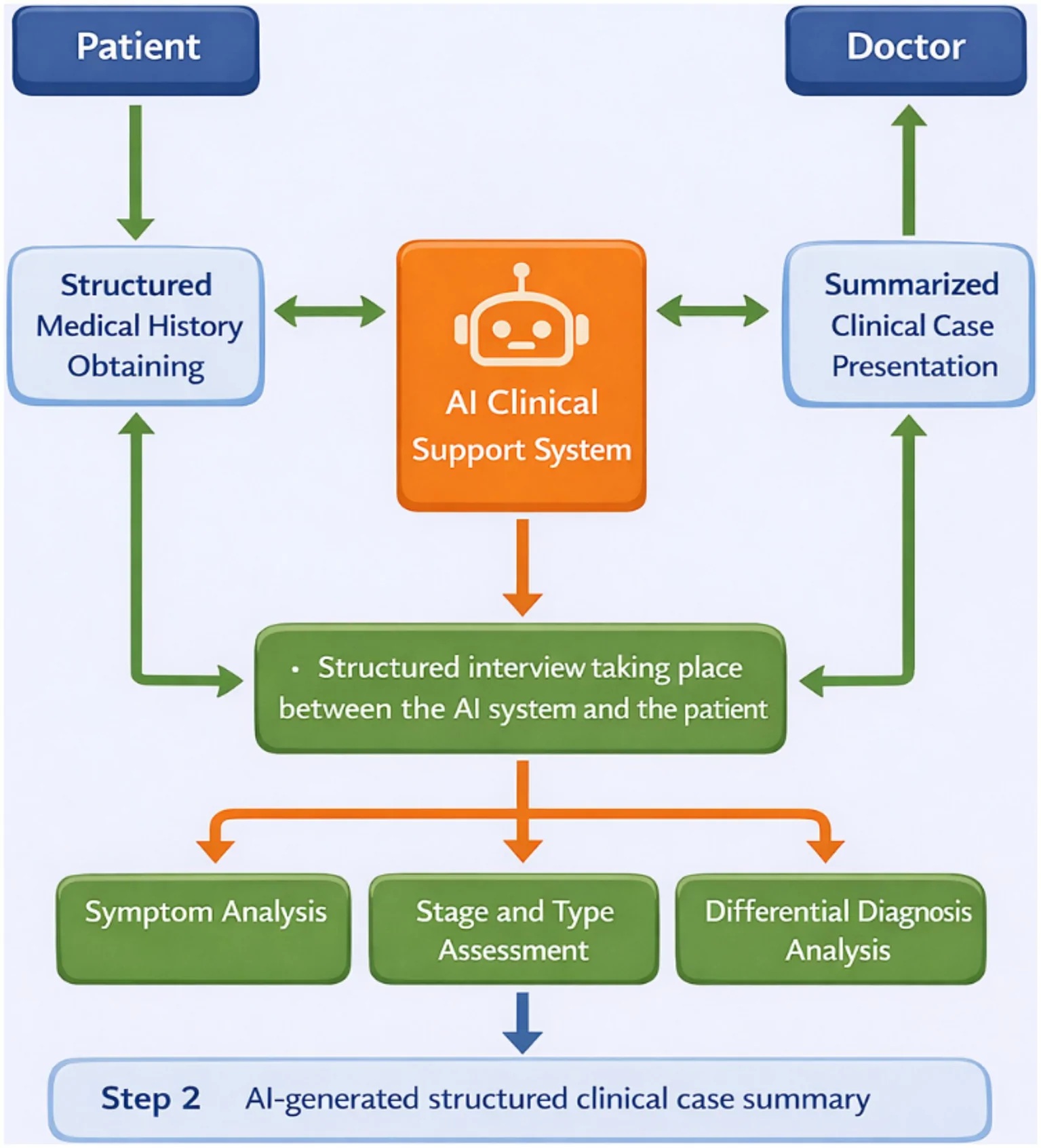
Schematic representation of LipoAssist-Physician interaction: from patient history taking and symptom evaluation to the generation of a structured clinical case summary.

In the initial stage, the artificial intelligence system interacts with the patient to obtain a structured medical history. During this phase, the system uses clear and understandable language to ensure patient comfort and asks questions in a structured, sequential manner. The interaction process between the AI system and the physician is illustrated in Figure 2. During the interview, the following clinical domains are systematically assessed:

- Chief complaint
- Onset and duration of symptoms
- Affected body regions
- Whether symptoms are bilateral and symmetrical
- Presence of pain, tenderness, heaviness, and easy bruising
- Presence or absence of foot involvement
- Relationship between weight changes, diet, and symptoms
- Family history
- Association with hormonal periods (e.g., puberty, pregnancy)

### 2.3 Scenario development process

A total of 10 simulated clinical scenarios were developed for use in this study. The scenarios were designed to represent commonly encountered clinical presentations in a lipedema outpatient clinic, as well as conditions included in the differential diagnosis. Accordingly, some scenarios were structured to reflect typical clinical features consistent with lipedema, while others were designed to represent differential diagnostic conditions such as obesity, lymphedema, and other causes of lower extremity swelling.

In the development of the scenarios, clinical characteristics, diagnostic criteria, and differential diagnostic pathways of lipedema were structured based on evidence from the literature. Each scenario was presented within a standardized framework including key clinical variables such as patient age, duration of symptoms, presence of pain, symmetry, relationship with weight changes, easy bruising, family history, and foot involvement. This approach enabled a systematic evaluation of the AI system’s performance across different clinical variations.

The scenarios were prepared in text format in a way that reflects clinical realism while maintaining standardization, and all scenarios were presented to the AI system using the same prompt structure. All clinical scenarios used in this study were entirely fictional and were created by the authors solely for research purposes. The scenarios were developed based on published diagnostic criteria, current literature, and clinical expertise to represent clinically plausible presentations of lipedema and its differential diagnoses. No real patients, medical records, identifiable clinical information, or patient-derived data were used in the preparation of these scenarios.

### 2.4 Evaluation of clinical features of lipedema

In patients with suspected lipedema, the system assesses the following clinical features:

- Bilateral and symmetrical fat distribution
- Pain or tenderness to pressure
- Easy bruising
- Persistence of fat distribution despite diet and exercise
- Relative sparing of the feet
- Limitation of mobility

Based on this information, the system collects data to assist in determining the possible stage and type of lipedema.

#### 2.4.1 Stages of lipedema

- Stage 1: The skin surface is smooth, but there is an increase in subcutaneous adipose tissue.
- Stage 2: The skin surface is irregular, with nodular adipose tissue.
- Stage 3: Marked tissue enlargement and significant deformities are present, and mobility may be notably impaired.

#### 2.4.2 Types of lipedema

- Type I – Hips
- Type II – Hips and thighs
- Type III – Entire legs
- Type IV – Arms
- Type V – Lower legs

#### 2.4.3 Differential diagnosis assessment

LipoAssist also collects data for differential diagnosis to identify conditions that may be confused with lipedema. In this context, the following conditions are evaluated:

- Obesity
- Lymphedema
- Chronic venous insufficiency
- Localized adiposity
- Lipolymphedema

For this purpose, the patient is also asked about the following:

- History of venous surgery or varicose vein treatment
- History of bariatric surgery
- Diagnosis or treatment of lymphedema
- Previous treatments for lipedema (e.g., compression therapy, physiotherapy, manual lymph drainage)

#### 2.4.4 General medical history

The system also collects the following information as part of the general medical history:

- Age
- Height and weight
- Recent weight changes
- Comorbidities
- Medications in use
- Previous surgeries
- Smoking and alcohol use

### 2.5 Case summary and physician presentation

After the patient interview is completed, the AI system presents the collected information in the form of a structured case summary. This presentation includes the following components:

- Patient demographic information
- Summary of symptoms
- Clinical findings
- Probable stage and type of lipedema
- Differential diagnosis assessment
- Suggested further clinical evaluations

This summary is intended to provide a rapid clinical overview prior to the physician’s face-to-face consultation with the patient.

### 2.6 Prompting

The system has been designed using a structured prompt engineering approach (13, 14). The prompt has been optimized to include role definition, step-by-step inquiry, and differential diagnosis guidance.

To maximize reproducibility, the same prompt was used for all simulated scenarios without modification. Each clinical scenario was initiated in a separate ChatGPT conversation to avoid contextual carry-over effects from previous interactions. No manual editing or regeneration of responses was performed after the initial AI output, and the first complete response generated by the model was retained for expert evaluation.

In GPT-based models, the *temperature* parameter is a key control mechanism that determines the diversity and randomness of generated responses. During interactions with patients, a *temperature* value of 0.7 was preferred. This setting enables the generation of more fluent, natural, and diverse responses. In interactions between the AI system and physicians, a *temperature* value of 0.4 was used. This level allows for more consistent, predictable, and reliable outputs. Except for the temperature setting, no additional model parameters were intentionally modified during the study. Default platform settings were retained for parameters such as response generation behavior.

The interactions were conducted using the ChatGPT web interface under the GPT-4 architecture available during the study period. As the ChatGPT interface does not provide user control over several inference parameters (e.g., top-p, maximum token limit, or internal model snapshot), these settings remained at the platform defaults throughout the study.

The Chain-of-Thought (CoT) approach is a technique that enables the model to structure its reasoning process step by step while generating responses (16). In this approach, expressions such as “Let us proceed step by step to reach the correct conclusion” can be used to guide the model’s reasoning. This approach aligns with zero-shot chain-of-thought prompting, allowing the model to perform logical inference without prior examples (17–19). Accordingly, the CoT approach was utilized to enhance the response accuracy and reasoning capability of the LipoAssist system.

No additional model customization, domain-specific training, external knowledge integration, or post-processing algorithms were applied beyond the predefined prompting strategy. The complete prompt is provided in the Supplementary materials to facilitate methodological transparency and future replication studies.

The objective of the present study was not to evaluate or modify the underlying GPT-4 model itself, but rather to assess the feasibility of a standardized prompt-engineering strategy designed for preliminary lipedema assessment. Therefore, all outputs generated by LipoAssist represent responses produced by GPT-4 operating under a predefined structured clinical workflow.

### 2.7 Expert evaluation and Likert analysis

The developed GPT-based system was named *LipoAssist* and presented with a unique avatar (Figure 2). The interaction process is initiated when the user selects the statement: “Hello, I am here because of my lipedema.”

After LipoAssist confirms its readiness in accordance with the defined prompt structure, 10 different simulated clinical cases were presented to the model. In each case, the artificial intelligence system posed sequential questions to the user; the process was advanced based on the user’s responses, and the resulting data were ultimately presented to three board-certified specialists in PM&R. The responses generated by GPT-4 were systematically analyzed using a Likert scale based on six distinct evaluation criteria (Table 1).

**Table 1:** Likert scale.

Criterion	1	2	3	4	5
Rating	Strongly disagree	Disagree	Neither agree nor disagree	Agree	Strongly agree
Criterion 1	The large language model (LLM) correctly understood and identified the problem.				
Criterion 2	The LLM correctly stated the most likely diagnosis.				
Criterion 3	The LLM provided appropriate recommendations for further diagnostic steps.				
Criterion 4	The LLM accurately assessed whether surgical intervention was necessary.				
Criterion 5	The LLM summarized the case effectively and presented it to the physician in a clear, comprehensible, and satisfactory manner.				
Criterion 6	The LLM obtained the patient history as thoroughly as I would have.				

The performance of the AI system developed in this study was independently evaluated by three board-certified specialists in Physical Medicine and Rehabilitation (PM&R) with clinical experience in the assessment and management of lipedema. Prior to the evaluation process, all reviewers received the same evaluation form together with standardized written instructions describing the six assessment criteria and the use of the 5-point Likert scale. Each evaluator independently reviewed all AI-generated case summaries without discussion with the other evaluators throughout the assessment process.

Formal evaluator blinding was not implemented because all reviewers were aware that the clinical summaries had been generated by the AI system. Similarly, no formal calibration session was conducted before scoring. To minimize potential evaluation bias, however, all reviewers used identical assessment criteria, evaluated the same scenarios independently, and completed their ratings without access to the evaluations of the other reviewers.

### 2.8 Statistical analysis

The data obtained in this study were analyzed using descriptive statistical methods. Although responses obtained using a 5-point Likert scale are ordinal by nature, mean and standard deviation values were calculated to summarize overall expert ratings across multiple scenarios and evaluation criteria. The use of parametric descriptive statistics for aggregated Likert-type data has been widely accepted in methodological literature when the primary objective is descriptive rather than inferential analysis (20–22). Accordingly, mean ± standard deviation values were calculated for each criterion and scenario, and the overall performance score of the system was determined by averaging all ratings.

Inter-rater reliability was assessed using the intraclass correlation coefficient (ICC), based on a two-way random-effects model with absolute agreement and average measures. ICC values were interpreted as follows: 0.00–0.50 indicating poor agreement, 0.50–0.75 moderate agreement, 0.75–0.90 good agreement, and values above 0.90 indicating excellent agreement. The combination of ICC and Kendall’s coefficient of concordance allowed assessment of inter-rater agreement using complementary approaches applicable to both aggregated numerical summaries and ordinal agreement.

To further evaluate agreement for ordinal Likert data, Kendall’s coefficient of concordance (Kendall’s W) was also calculated.

Data were presented as mean ± standard deviation. Given the exploratory and proof-of-concept nature of the study, no formal hypothesis testing was performed. All statistical analyses were conducted using IBM SPSS Statistics (Version 27.0; IBM Corp., Armonk, NY, USA).

## 3 Results

The performance of the LipoAssist system was evaluated by three board-certified specialists in PM&R using a Likert scale across 10 simulated clinical scenarios and 6 assessment criteria. A total of 180 ratings were analyzed. The overall mean performance score of the system was found to be 3.55 ± 0.64.

In the criterion-based evaluation, the highest mean scores were obtained for accurate understanding of the clinical condition (4.63 ± 0.49) and correct identification of the most likely diagnosis (4.53 ± 0.51). The lowest scores were observed in the areas of advanced diagnostic recommendations (2.17 ± 0.83) and assessment of surgical necessity (1.93 ± 0.64). The clarity of case summary presentation (3.87 ± 0.63) and adequacy of patient history taking (3.73 ± 0.64) were rated at a moderate-to-good level (Table 2).

**Table 2:** Mean scores and standard deviations according to evaluation criteria.

Criterion	Average	Standard deviation
Criterion 1	4.63	0.49
Criterion 2	4.53	0.51
Criterion 3	2.17	0.83
Criterion 4	1.93	0.64
Criterion 5	3.87	0.63
Criterion 6	3.73	0.64

In the scenario-based evaluation, mean scores were found to range between 3.17 ± 0.62 and 3.78 ± 0.67. The highest mean score was observed in Scenario 10 (3.78 ± 0.67), whereas the lowest mean score was obtained in Scenario 4 (3.17 ± 0.62). The relatively low standard deviation values across scenarios indicate a consistent level of agreement among the evaluators (Table 3).

**Table 3:** Mean scores and standard deviations by scenario.

Scenario	Average	Standard deviation
Scenario 1	3.61	0.51
Scenario 2	3.39	0.49
Scenario 3	3.39	0.67
Scenario 4	3.17	0.62
Scenario 5	3.61	0.51
Scenario 6	3.56	0.76
Scenario 7	3.39	0.67
Scenario 8	3.72	0.70
Scenario 9	3.56	0.76
Scenario 10	3.78	0.67

Overall, the findings indicate that the GPT-4–based AI system demonstrates strong performance in structured patient history taking and interpretation of the clinical condition; however, its performance is comparatively lower in more advanced clinical decision-making domains, such as diagnostic recommendations and assessment of surgical necessity.

Inter-rater reliability analysis demonstrated good agreement among evaluators, with an intraclass correlation coefficient (ICC) of 0.82 (95% CI: 0.68–0.91). Additionally, Kendall’s coefficient of concordance (Kendall’s W) was 0.79, indicating a high level of consistency (Table 4).

**Table 4:** Inter-rater reliability analysis.

Analysis	Value	95% Confidence interval	Interpretation
ICC	0.82	0.68–0.91	Good agreement
Kendall’s W	0.79	—	High agreement

## 4 Discussion

This study evaluated the performance of a GPT-4–based AI clinical assistant developed for the preliminary assessment of lipedema, using simulation-based clinical scenarios. The overall mean score of 3.55 ± 0.64 indicates that the system demonstrates satisfactory performance, particularly in accurately understanding the clinical condition and identifying the most likely diagnosis. However, its performance was lower in more advanced clinical decision-making processes, such as diagnostic recommendations and assessment of surgical necessity. These findings are consistent with previous studies reporting that LLMs perform well in history taking and organization of clinical information but are more limited in complex diagnostic and therapeutic decision-making (8–11, 23, 24). Compared with previous simulation-based studies, the present study provides a structured criterion-based evaluation of a GPT-4–based clinical workflow across multiple clinically representative scenarios. The findings therefore reflect the performance of a standardized prompt-engineered workflow built upon GPT-4 rather than validation of a newly developed artificial intelligence model. An additional consideration is the rapidly evolving nature of commercial large language models. Because GPT models are periodically updated, identical prompts may not always produce identical outputs over time, limiting long-term reproducibility. Accordingly, the reported scores should be interpreted as expert evaluations of AI-generated clinical summaries rather than objective measures of diagnostic accuracy. Future studies using API-based implementations with fixed model versions may further improve methodological reproducibility.

The number of studies evaluating GPT-4 in the context of lipedema remains limited. In a simulation-based study by Leypold et al. (15), GPT-4 demonstrated a higher mean performance score (4.24), particularly in patient history taking and case summarization, while showing relatively lower performance in advanced diagnostic and surgical decision-making. These findings are consistent with the present study. However, the current study contributes additional evidence by including a different specialist group (PM&R physicians) and a broader range of clinical scenarios.

The high scores observed in understanding the clinical condition and identifying the most likely diagnosis highlight the strengths of GPT-4 in natural language processing and clinical data structuring. The system was able to systematically assess symptoms associated with lipedema and organize them into a clinically meaningful framework. This suggests that AI-assisted tools may be particularly valuable in conditions such as lipedema, where diagnosis heavily relies on detailed anamnesis (1, 4–6). Furthermore, structured presentation of patient data may facilitate a more efficient and informed clinical evaluation.

In contrast, the lowest scores were observed in advanced diagnostic recommendations and assessment of surgical necessity. One possible explanation is that the AI system relies exclusively on patient-reported history and does not incorporate physical examination findings. However, the diagnosis and management of lipedema typically require a comprehensive, multidisciplinary evaluation, including physical examination, imaging, and clinical expertise (3, 6). Therefore, tasks such as advanced diagnostic planning and surgical decision-making inherently require higher-level clinical reasoning.

Additionally, these decisions depend on multiple interacting factors, including disease stage, functional impairment, response to conservative treatment, and patient expectations. Such multidimensional decision-making processes, which require contextual interpretation and experience-based judgment, remain challenging for LLMs (9, 10). Moreover, the lack of training on disease-specific clinical algorithms may further contribute to these limitations (12).

The relatively narrow range of scenario-based scores suggests that the system demonstrated consistent performance across different clinical contexts. While this indicates robustness, it may also reflect the standardized and simulation-based design of the study. This consistency was further supported by the inter-rater reliability analysis.

Inter-rater reliability in this study was found to be at a good level (ICC = 0.82; 95% CI: 0.68–0.91; Kendall’s W = 0.79). These values fall within the upper range compared to inter-rater reliability (IRR) results reported in clinical evaluation studies involving LLMs. In the literature, studies with similar methodologies have reported ICC values ranging from moderate to good levels. For instance, in studies evaluating AI-based clinical decision support systems, ICC values have been reported between 0.54 and 0.87 (25). In another study assessing the performance of a GPT-based clinical assistant, the overall ICC value was found to be 0.77 (26). Conversely, lower agreement levels (ICC ≈ 0.25–0.65) have also been reported in domain-specific evaluations (27, 28). In the present study, the ICC value of 0.82, calculated based on average measures, indicates strong consistency among evaluators and suggests that the outputs generated by the LLM are interpreted similarly by different experts. However, direct comparisons across studies should be interpreted with caution, as ICC estimates may vary depending on the statistical model, number of evaluators, scoring systems, and evaluation criteria. Although descriptive statistics based on mean values were used to summarize expert ratings, agreement between evaluators was additionally assessed using both ICC and Kendall’s W to provide complementary measures of reliability. This combined approach has been recommended in studies evaluating expert agreement for ordinal rating scales (20–22).

The findings of this study indicate that LLMs hold significant potential in clinical practice, particularly in the standardized collection of patient history and the organization of clinical information (8–11). In busy outpatient settings, an AI-assisted system that conducts a preliminary interview and presents structured data may contribute to a more efficient use of physician–patient consultation time. Additionally, standardized anamnesis may reduce the risk of overlooking critical clinical information. Nevertheless, the present findings indicate that such systems should function as adjunctive decision-support tools under physician supervision rather than as autonomous clinical decision-makers.

However, the results also indicate that it is not appropriate to use AI systems as independent clinical decision-makers. Particularly in high-level clinical decision domains such as advanced diagnostic recommendations and the assessment of surgical necessity, physician evaluation remains indispensable (23, 24). Therefore, LLMs should be positioned not as replacements for clinical decision-making, but as supportive tools that assist physician judgment.

Beyond diagnostic performance, several broader challenges should be considered before integrating large language models into routine clinical practice. One of the most important concerns is the possibility of hallucinations, whereby AI systems may generate factually incorrect or unsupported clinical statements that appear plausible. Such outputs may create substantial risks if accepted uncritically in patient care, particularly in rare or diagnostically challenging disorders such as lipedema. Consequently, AI-generated recommendations should always be interpreted within the context of physician oversight rather than being regarded as independent clinical decisions (7, 20, 29, 30).

Another important consideration is explainability. Unlike conventional rule-based clinical decision support systems, large language models do not explicitly reveal the reasoning process underlying their recommendations. This limited transparency may reduce clinician trust, complicate error detection, and make clinical validation more difficult. Accordingly, AI systems intended for healthcare should prioritize transparent reporting of their intended role, methodological limitations, and evaluation procedures (31, 32).

Automation bias also represents a potential challenge. Previous research has demonstrated that clinicians may unintentionally place excessive confidence in AI-generated recommendations, particularly when outputs appear coherent and authoritative. Therefore, AI-assisted systems should be viewed as decision-support tools that complement, rather than replace, physician judgment. Clinical responsibility should remain entirely with the treating physician, who must critically evaluate AI-generated outputs together with the patient’s history, physical examination findings, and other diagnostic information (7, 20, 33).

Finally, the implementation of AI systems in healthcare should be accompanied by careful consideration of ethical principles, patient safety, data governance, and evolving regulatory frameworks. Although the present study did not involve real patient data, future clinical validation studies should ensure compliance with applicable ethical standards and regulatory requirements before implementation in routine healthcare settings (34–36).

Despite these limitations, the present study has several notable strengths. To the best of our knowledge, this is among the first studies to systematically evaluate a structured GPT-4–based clinical workflow specifically designed for the preliminary assessment of lipedema. The use of standardized simulated scenarios, predefined evaluation criteria, independent expert assessment, and complementary inter-rater reliability analyses provides a transparent methodological framework that may facilitate future multicenter validation studies involving real patients and comparative evaluations against alternative AI systems.

This study has several limitations that should be considered when interpreting the findings. First, the evaluation was conducted exclusively using simulated clinical scenarios rather than real patient encounters. Consequently, the present study should be regarded as a proof-of-concept investigation under controlled simulation conditions rather than a clinical validation study. The findings do not provide evidence regarding diagnostic accuracy, clinical validity, or real-world applicability of the proposed system. Prospective validation using real patients is required before any conclusions regarding routine clinical implementation can be drawn. Because the scenarios were fictional rather than patient-derived, future studies should evaluate the proposed workflow using prospectively collected real-world clinical data.

Second, although the scenarios were designed to represent clinically relevant presentations commonly encountered in lipedema outpatient practice, several scenarios intentionally included characteristic clinical findings to ensure standardized assessment across evaluators. This design may have facilitated diagnostic reasoning by the large language model and therefore may have resulted in an overestimation of its performance compared with real-world clinical encounters, where symptom presentation is often more heterogeneous and ambiguous.

Third, the scenarios were limited in number and cannot fully represent the wide spectrum of clinical variability observed in patients with lipedema and related disorders. Although efforts were made to improve clinical realism, simulation-based assessment cannot fully reproduce the complexity, uncertainty, and variability of routine clinical practice. Furthermore, the evaluation involved only three PM&R specialists, and formal evaluator blinding and calibration procedures were not implemented, although standardized assessment criteria and independent scoring were used to minimize potential bias.

Another important limitation is the absence of an external reference standard. The performance of LipoAssist was not compared with physician-generated assessments, established lipedema clinical guidelines, or alternative large language models. Consequently, although expert ratings provide valuable information regarding the perceived quality of AI-generated outputs, they do not allow direct comparison with existing diagnostic approaches or other AI systems. Future studies should incorporate comparative benchmark analyses using physician assessments, guideline-based evaluations, and multiple large language models to better contextualize system performance.

Finally, the cross-sectional design of the study did not allow for assessment of the real-time integration of the AI system into clinical workflows or its impact on patient outcomes. Future studies incorporating real patient data, prospective validation designs, and multicenter expert evaluations are essential to establish the clinical validity and real-world applicability of such systems.

## 5 Conclusion

This proof-of-concept study demonstrated that a GPT-4–based AI system showed promising performance in the preliminary assessment of simulated lipedema cases, particularly in structured patient history taking, interpretation of clinical findings, and identification of the most likely diagnosis. However, these findings were obtained exclusively under simulated conditions and should not be interpreted as evidence of clinical validity or diagnostic accuracy in real-world practice.

Accordingly, the proposed approach should currently be considered an experimental decision-support strategy rather than a clinically validated diagnostic tool. Future prospective studies involving real patients, multicenter validation, and comparisons with physician performance are required before considering integration into routine clinical workflows.

Future research incorporating real patient data, a broader range of clinical scenarios, and multicenter expert evaluations is needed to further elucidate the clinical applicability, reliability, and potential integration of these systems into routine practice.

## Data Availability

The original contributions presented in the study are included in the article/Supplementary material, further inquiries can be directed to the corresponding author.

## References

[1] Wold LE Hines EA Jr Allen EV. Lipedema of the legs: a syndrome characterized by fat legs and edema. Ann Intern Med. (1951) 34:1243–50. doi: 10.7326/0003-4819-34-5-1243, 14830102

[2] Herbst KL. Rare adipose disorders (RADs) masquerading as obesity. Acta Pharmacol Sin. (2012) 33:155–72. doi: 10.1038/aps.2011.153, 22301856 PMC4010336

[3] Forner-Cordero I Szolnoky G Forner-Cordero A Kemény L. Lipedema: an overview of its clinical manifestations, diagnosis and treatment of the disproportional fatty deposition syndrome – systematic review. Clin. Obes. (2012) 2:86–95. doi: 10.1111/j.1758-8111.2012.00045.x 25586162

[4] Al-Ghadban S Cromer W. Lipedema: a painful adipose tissue disorder. J Clin Med. (2019) 8:E659. doi: 10.3390/jcm8050659

[5] Langendoen SI Habbema L Nijsten TE. Lipedema: from clinical presentation to therapy. J Am Acad Dermatol. (2009) 63:722–4. doi: 10.1016/j.jaad.2009.06.026, 19785610

[6] Dadras M Mallinger PJ Corterier CC Theodosiadi S Ghods M. Lipedema: diagnostic and management challenges. Int J Women's Health. (2017) 9:389–95. doi: 10.2147/IJWH.S130078

[7] Topol EJ. High-performance medicine: the convergence of human and artificial intelligence. Nat Med. (2019) 25:44–56. doi: 10.1038/s41591-018-0300-7, 30617339

[8] Esteva A Kuprel B Novoa RA Ko J Swetter SM Blau HM . A guide to deep learning in healthcare. Nat Med. (2019) 25:24–9. doi: 10.1038/s41591-018-0316-z 30617335

[9] Rajkomar A Dean J Kohane I. Machine learning in medicine. N Engl J Med. (2019) 380:1347–58. doi: 10.1056/NEJMra1814259, 30943338

[10] Bommas-Ebert U Brönnimann A Huwendiek S. ChatGPT and GPT-4 in medical education and clinical practice: a systematic review. Med Educ. (2023) 57:1047–55. doi: 10.1111/medu.15160

[11] Sallam M. ChatGPT utility in healthcare education, research, and practice: systematic review. Healthcare (Basel). (2023) 11:887. doi: 10.3390/healthcare11060887 36981544 PMC10048148

[12] OpenAI. (2023) GPT-4 technical report. arXiv. 2023, arXiv:2303.08774. Available online at: https://arxiv.org/abs/2303.08774 (Accessed April 17, 2026).

[13] Kojima T Gu SS Reid M Matsuo Y Iwasawa Y. Large Language Models are Zero-Shot Reasoners. arXiv:2205.11916 (2022). doi: 10.48550/arXiv.2205.11916

[14] White J Fu Q Hays S Sandborn M Olea C Gilbert H . A Prompt Pattern Catalog to Enhance Prompt Engineering with ChatGPT. arXiv arXiv:2302.11382 (2023). doi: 10.48550/arXiv.2302.11382

[15] Leypold T Lingens LF Beier JP Boos AM. Integrating AI in lipedema management: assessing the efficacy of GPT-4 as a consultation assistant. Life. (2024) 14:646. doi: 10.3390/life14050646, 38792666 PMC11122530

[16] Wei J Wang X Schuurmans D Bosma M Xia F Chi E . Chain-of-thought prompting elicits reasoning in large language models. Adv Neural Inf Process. Syst. (2022) 35:24824–37. doi: 10.52202/068431-1800

[17] Wei J. Bosma M. Zhao V.Y. Guu K. Yu A.W. Lester B. . Finetuned Language Models Are Zero-Shot Learners. International Conference on Learning Representations (ICLR), (2022) arXiv:2109.01652.

[18] Kojima T Gu SS Reid M Matsuo Y Iwasawa Y. Large language models are zero-shot reasoners. Adv Neural Inf Process Syst. (2022) 35:22199–213. doi: 10.52202/068431-1613

[19] Mesko B. Prompt engineering in medical AI. NPJ Digit Med. (2023) 6:75. doi: 10.1038/s41746-023-00801-737100871

[20] Norman G. Likert scales, levels of measurement and the “laws” of statistics. Adv Health Sci Educ Theory Pract. (2010) 15:625–32. doi: 10.1007/s10459-010-9222-y, 20146096

[21] Sullivan GM Artino AR Jr. Analyzing and interpreting data from Likert-type scales. J Grad Med Educ. (2013) 5:541–2. doi: 10.4300/JGME-5-4-18, 24454995 PMC3886444

[22] Carifio J Perla RJ. Resolving the 50-year debate around using and misusing Likert scales. Med Educ. (2008) 42:1150–2. doi: 10.1111/j.1365-2923.2008.03172.x, 19120943

[23] Patel SB Lam K. ChatGPT in clinical decision making. Lancet Digit Health. (2023) 5:e376–7. doi: 10.1016/S2589-7500(23)00096-5

[24] Kung TH Cheatham M Medenilla A Sillos C De Leon L Elepaño C . Performance of ChatGPT on USMLE: potential for AI-assisted medical education using large language models. PLoS Digit Health. (2023) 2:e0000198. doi: 10.1371/journal.pdig.0000198, 36812645 PMC9931230

[25] Wang T Chen R Wang B Zou C Fan N Yuan S . Evaluating large language model performance on WHO surgical site infection prevention guidelines using expert panel scoring. J Med Internet Res. (2025) 27:e75567. doi: 10.2196/75567 40744114 PMC12313333

[26] Zhang Y Liu H Chen X Wang R Li Q. Development and evaluation of a GPT-based outpatient clinical assistant using expert Likert scoring: inter-rater reliability analysis. JMIR Med Inform. (2025) 13:e68980. doi: 10.2196/68980 40705609 PMC12288767

[27] Dietrich LG De Pellegrin L Rinaldi V Harder Y Vögelin E Rothenfluh E. Diagnostic reasoning performance of large language models in hand surgery: an expert panel evaluation. J Clin Med. (2025) 14:8045. doi: 10.3390/jcm14228045 41303080 PMC12653297

[28] Seo J Choi D Kim T Cha WC Kim M Yoo H . Reliability of large language model–generated clinical documentation using expert evaluation framework. J Med Internet Res. (2024) 26:e58329. doi: 10.2196/58329 39566044 PMC11618017

[29] World Health Organization. Ethics and Governance of artificial Intelligence for Health: WHO Guidance. Geneva: World Health Organization (2021).

[30] Mesko B Topol EJ. The role of generative artificial intelligence in healthcare. NPJ Digit Med. (2023) 6:120. doi: 10.1038/s41746-023-00873-0 37414860 PMC10326069

[31] Markus AF Kors JA Rijnbeek PR. The role of explainability in creating trustworthy artificial intelligence for health care: a comprehensive survey of the terminology, design choices, and evaluation strategies. J Biomed Inform. (2021) 113:103655. doi: 10.1016/j.jbi.2020.103655, 33309898

[32] Chen RJ Wang JJ Williamson DFK Chen TY Lipkova J Lu MY . Algorithm fairness in artificial intelligence for medicine and healthcare. Nat Biomed Eng. (2023) 7:719–42. doi: 10.1038/s41551-023-01056-8 37380750 PMC10632090

[33] Sutton RT Pincock D Baumgart DC Sadowski DC Fedorak RN Kroeker KI. An overview of clinical decision support systems: benefits, risks, and strategies for success. NPJ Digit Med. (2020) 3:17. doi: 10.1038/s41746-020-0221-y, 32047862 PMC7005290

[34] European Union. Regulation (EU) 2024/1689 of the European Parliament and of the Council laying down harmonised rules on artificial intelligence (Artificial Intelligence Act). Off J Eur Union. (2024)

[35] U.S. Food and Drug Administration. Artificial Intelligence/Machine Learning (AI/ML)-Based Software as a Medical Device (SaMD) Action Plan. Silver Spring, MD: FDA (2021).

[36] World Health Organization. Regulatory Considerations on Artificial Intelligence for Health. Geneva: World Health Organization (2023).

