# Supplementary material for: LipoAssist: a structured GPT-4–based clinical workflow for preliminary lipedema assessment
Source: Front Med (Lausanne). 2026 Aug 31;13:1909592. doi: 10.3389/fmed.2026.1909592 (PMC13572100; doi:10.3389/fmed.2026.1909592)
Supplement: Supplementary file 1 [file Supplementary_file_1.docx]

**Supplementary materials: Scenarios**

**Scenario 1**

A 35-year-old female patient presented with complaints of long-standing, symmetrical thickening in both legs accompanied by pain that increases with pressure. It was learned that the symptoms began after puberty and progressively worsened over time. The areas below the knees and the feet were reported to be normal. The patient also stated that she experiences easy bruising and that the thickness of her legs did not change despite weight loss. It was noted that her mother has similar complaints.

**Scenario 2**

A 42-year-old female patient presented with complaints of generalized weight gain and thickening of the legs over the past three years. It was noted that there was also prominent fat accumulation in the abdominal and facial regions, with no associated pain or tenderness. The patient reported that after losing ten kilograms, a noticeable reduction in leg thickness was observed. No swelling of the feet was reported.

**Scenario 3**

A 50-year-old female patient presented with a complaint of marked swelling in the right leg. The swelling was noted to be unilateral, involving the dorsum of the foot, and pitting on pressure. The patient reported minimal pain. It was also noted that she had a history of knee prosthesis surgery.

**Scenario 4**

A 48-year-old female patient presented with complaints of symmetrical thickening of both legs and progressively increasing swelling around the ankles in recent years. The patient reported associated pain and a sensation of heaviness. Her body mass index (BMI) was 32. It was noted that the use of compression therapy provided partial relief.

**Scenario 5**

A 28-year-old female patient presented with complaints of a feeling of fullness in the thighs and mild tenderness. The skin surface was noted to be smooth, and the patient reported occasional easy bruising. Her body mass index (BMI) was within normal limits, and no relevant family history was identified.

**Scenario 6**

A 52-year-old female patient presented with complaints of marked bilateral increase in leg volume and difficulty walking. Nodular changes were observed on the skin surface, and the patient reported pain and easy bruising. Her body mass index (BMI) was 35.

**Scenario 7**

A 33-year-old female patient presented with complaints of leg thickening and pain that began after her second pregnancy. The symptoms were reported to have become more pronounced in the postpartum period. It was noted that the feet were not affected and that the complaints did not change with dietary measures.

**Scenario 8**

A 37-year-old female patient presented with complaints of fat accumulation limited to the hip region. The condition was noted to be symmetrical, with no associated pain, tenderness, or easy bruising. The patient reported that the hip region decreased in size with diet. The feet were described as normal.

**Scenario 9**

A 41-year-old female patient presented with complaints of thickening in the legs and upper arms. The patient reported associated pain and easy bruising. It was noted that the feet were not affected and that her mother had similar complaints.

**Scenario 10**

A 55-year-old female patient presented with complaints of leg swelling that increases throughout the day. The swelling was reported to worsen in the evening and improve in the morning. The patient had a history of varicose veins, and the swelling was noted to be pitting on pressure, with involvement of the feet.
